# The Effect of the Duration of Feeding a Diet Containing Cuban Cricket (*Gryllus assimilis*) Meal on Fecal Bacterial Enzyme Activity and Nutrient Digestibility in Adult Rabbits

**DOI:** 10.3390/ani16162619

**Published:** 2026-08-21

**Authors:** Janusz Strychalski, Jerzy Juśkiewicz, Andrzej Gugołek

**Affiliations:** 1 Department of Fur-Bearing Animal Breeding and Game Management, University of Warmia and Mazury in Olsztyn, Oczapowskiego 2, 10-719 Olsztyn, Poland; gugolek@uwm.edu.pl; 2 InLife Institute of Animal Reproduction and Food Research, Polish Academy of Sciences, Trylińskiego 18, 10-683 Olsztyn, Poland

**Keywords:** Cuban cricket meal, fecal bacterial enzyme activity, apparent digestibility, detergent fiber fractions, rabbit nutrition

## Abstract

Insect-derived feed ingredients are increasingly considered alternative protein sources, but their time-dependent effects on digestive function remain unclear. This study evaluated how the duration of feeding a diet containing Cuban cricket meal (CCM) affected fecal bacterial enzyme activity and apparent nutrient digestibility in adult rabbits. Thirty-two New Zealand White male rabbits were allocated to four groups. The control group received a diet containing 14% soybean meal throughout the six-week trial, whereas experimental groups received soybean meal followed by a diet in which 10% CCM replaced soybean meal for the final two, four, or six weeks. It was found that the fecal enzymatic response depended on feeding duration. Short-term feeding induced limited changes, whereas longer feeding periods (four and six weeks) increased the activity of enzymes involved in the degradation of complex substrates, including chitin, and enhanced the extracellular activity of several enzymes. Digestibility of dry matter, crude protein, ether extract, and energy was unaffected. By contrast, neutral detergent fiber, acid detergent fiber, and acid detergent lignin digestibility increased after four and six weeks (*p* < 0.001). These results could be interpreted as indirect evidence that rabbits’ gut microbiota requires an adaptation period to effectively respond to insect-derived components.

## Simple Summary

Insects are increasingly being considered as an alternative protein source in animal nutrition. However, their effects on gut function, especially in terms of how quickly animals adapt to such diets, are still unclear. This study examined whether the duration of feeding adult rabbits a diet containing Cuban cricket meal influenced fecal bacterial enzyme activity and apparent nutrient digestibility. Short-term feeding (two weeks) did not result in noticeable changes in fiber utilization, whereas longer feeding periods (four and six weeks) led to clear increases in the activity of enzymes involved in degrading structural carbohydrates, including chitin. This was accompanied by improved digestibility of fiber fractions. These results could be interpreted as indirect evidence that rabbits’ gut microbiota requires an adaptation period to effectively respond to insect-derived components, and that this response is linked to changes in microbial enzyme activity rather than to nutrient digestion itself.

## 1. Introduction

One of the key challenges facing contemporary agriculture and the food production sector is ensuring an adequate protein supply to meet the demands of the growing global population [1,2]. In response to this challenge, recent years have witnessed an increased interest in the use of insects as a protein source in animal nutrition [3]. Insects are characterized by a fast growth rate and high reproductive potential. Approximately 2 kg of feed are needed to produce 1 kg of insect biomass. Insect meals have high energy content, with protein levels ranging from 40% to 60% depending on the species and developmental stage [4]. There is growing evidence to indicate that the effects of insect meals on animals extend beyond nutritional aspects. These meals can also significantly affect gastrointestinal function, including fermentation processes in the large intestine [5].

Chitin, a structural polysaccharide that forms the exoskeleton of insects, is a characteristic component of insect meals. In mammals, chitin is primarily degraded by gut microbiota. Therefore, the efficiency of chitin utilization is determined by the capacity of gut microorganisms to synthesize chitinolytic enzymes [6]. Chitin is predominantly determined analytically as acid detergent fiber (ADF) and, to a lesser extent, as the difference between ADF and acid detergent lignin (ADL). However, chitin is an insect (or fungal) aminopolysaccharide, and its basic unit is N-acetylglucosamine [7,8]. Therefore, the mechanism of its degradation in the large intestine differs from that of lignocellulose. It is believed that the presence of chitin in the diet may induce specific changes in the enzyme activity of gut microorganisms, thereby modifying fermentation processes [9].

It has been demonstrated that insect meals can be added to the diets of various livestock species, including poultry [10], pigs [11], cattle [12], sheep [13], and rabbits. Previous studies investigating insect meals in rabbit nutrition have focused primarily on performance parameters, selected biochemical indicators, and the health status of animals [14,15,16]. However, the extent to which the gut microbiota of rabbits adapt to the presence of chitin in their diet depending on the duration of its administration remains to be determined. It is unclear whether changes in fecal bacterial enzyme activity are detectable after short-term exposure to insect meal, or whether measurable responses become evident only after a longer feeding period. This is particularly important with regard to chitinolytic enzymes and enzymes involved in the degradation of structural fiber fractions, as their activity may directly affect the digestibility of neutral detergent fiber (NDF), ADF, and ADL [17,18].

The objective of this study was to investigate how the duration of the dietary inclusion of Cuban cricket meal (CCM) is associated with changes in fecal bacterial enzyme activity. In addition, the study aimed to assess whether these changes coincide with measurable differences in the apparent digestibility of nutrients, with particular emphasis on NDF, ADF, and ADL and their contribution to overall nutrient utilization and energy efficiency.

## 2. Materials and Methods

### 2.1. Experimental Factor

The experimental factor was the duration of feeding a diet containing CCM to rabbits. Two nutritionally balanced pelleted diets were formulated: a control diet containing 14% soybean meal (SBM) as the main protein source, and an experimental diet in which SBM was completely excluded and replaced with 10% CCM (Table 1). Both diets were formulated to be isonitrogenous and isoenergetic (Table 2). The feeding trial lasted six weeks and included four groups. Control group (C) rabbits were fed an SBM-based diet throughout the entire six-week experiment. The experimental groups were administered the following diets: group CCM2wk—an SBM-based diet for four weeks, followed by a CCM diet for two weeks, group CCM4wk—an SBM-based diet for two weeks, followed by a CCM diet for four weeks, and group CCM6wk—a CCM diet for six weeks.

The 2-, 4-, and 6-week CCM feeding periods were selected to represent short-term, intermediate, and full-experiment exposure to the insect meal within the same six-week trial. The shortest period exceeded the adaptation period commonly used in rabbit digestibility studies, whereas the longer periods allowed evaluation of whether fecal bacterial enzyme activity and apparent nutrient digestibility changed with prolonged exposure to a chitin-containing ingredient. The study was designed as a parallel-group endpoint comparison, with fecal samples collected once from all animals at 32 weeks of age to ensure that the groups were evaluated at the same age. Therefore, intermediate fecal samples were not collected from the CCM6wk group at weeks 2 and 4. The design was not intended to determine within-animal temporal trajectories of enzymatic activity or digestibility.

### 2.2. Animals and Housing

Thirty-two New Zealand White male rabbits raised in a closed facility on a farm in northern Poland were used in the experiment. The animals were randomly allocated to four equal groups (*n* = 8), comparable in terms of origin and initial body weight. The mean initial body weights were 4.05 ± 0.24, 4.03 ± 0.21, 4.07 ± 0.23, and 4.04 ± 0.22 kg in the control, CCM2wk, CCM4wk, and CCM6wk groups, respectively. The experiment began when rabbits were 26 weeks of age and ended at 32 weeks of age. Rabbits were housed individually in wire-net flat-deck cages (0.5 × 0.6 × 0.4 m). Standard environmental conditions were maintained throughout the experiment: ambient temperature of 18–20 °C, relative humidity of 60–75%, intensive ventilation, and a controlled photoperiod of 16 h light and 8 h darkness.

Animals were fed pelleted diets ad libitum and had free access to water supplied through nipple drinkers. Cecotrophy was allowed in all groups.

### 2.3. Experimental Procedures

During the last eight days of the experiment, feces were collected daily for apparent digestibility calculations and bacterial enzyme activity assays. First, a seven-day digestibility-balance trial was conducted in the same cages adapted for total fecal collection. Non-ingested feed residues and feces intended for apparent digestibility calculations were collected daily and weighed with an accuracy of 1 g. The collected feces were frozen, and samples of feces and diets were subsequently dried at 60 °C and ground for chemical analyses. Apparent nutrient digestibility coefficients (DC) were calculated using the balance method according to the formula:

DC = (*a* − *b*)/*a* × 100%,

where *a* is the nutrient content of feed and *b* is the nutrient content of feces.

The day after the completion of fecal collection for the digestibility assay, fecal samples intended for bacterial enzyme activity assays were collected separately from freshly voided hard feces at the common terminal sampling point. The area beneath each cage was inspected at approximately 30-min intervals, so samples were collected within approximately 30 min of defecation. Only clearly formed hard fecal pellets were collected, and no soft feces (cecotropes) were observed in the collection area during sampling. They were immediately placed on ice and stored at −70 °C until analysis. Before the assays, fecal material was thawed on ice and prepared as a 1:10 (*v*/*v*) dilution in 100 mM phosphate buffer, pH 7.0, as described below.

### 2.4. Chemical Analyses

All analyses were carried out in duplicate.

The chemical composition of feed and feces was determined by the official methods of analysis of AOAC International [19]. Dry matter (DM) content was determined in a laboratory drier, at 103 °C. Crude ash (CA) content was estimated by sample mineralization in a muffle furnace (Czylok Sp. z o.o., Jastrzębie-Zdrój, Poland) at 600 °C. Total nitrogen content was determined by the Kjeldahl method, in the FOSS TECATOR Kjeltec 2200 Auto Distillation Unit (FOSS Tecator AB, Höganäs, Sweden). Ether extract (EE) content was estimated by the Soxhlet method, in the FOSS SOXTEC SYSTEM 2043 (FOSS Tecator AB, Höganäs, Sweden).

Both NDF and ADF were analyzed in the FOSS TECATOR Fibertec 2010 System (FOSS Tecator AB, Höganäs, Sweden); NDF was determined as described by Van Soest et al. [20], and ADF was determined using the official methods of analysis of AOAC International [19]. Gross energy content was determined in a bomb calorimeter (IKA^®^ C2000 basic, Staufen, Germany).

Fecal samples were analyzed to determine the activity of selected bacterial enzymes (α- and β-glucosidase, α- and β-galactosidase, β-glucuronidase, α-arabinopyranosidase, α-arabinofuranosidase, β-xylosidase, β-cellobiosidase, *N*-acetyl-β-D-glucosaminidase—NAGase, and β-chitobiosidase), which was measured as the rate of release of p-nitrophenol (PNP) or o-nitrophenol (ONP) from the respective nitrophenyl glucosides (Merck Life Science Sp. z o.o., Poznań, Poland), according to a previously described method [14]. The following substrates were used: p-nitrophenyl-α-D-glucopyranoside (for α-glucosidase), p-nitrophenyl-β-D-glucopyranoside (for β-glucosidase), p-nitrophenyl-α-D-galactopyranoside (for α-galactosidase), o-nitrophenyl-β-D-galactopyranoside (for β-galactosidase), p-nitrophenyl-β-D-glucuronide (for β-glucuronidase), p-nitrophenyl-α-L-arabinopyranoside (for α-arabinopyranosidase), p-nitrophenyl-α-L-arabinofuranoside (for α-arabinofuranosidase), p-nitrophenyl-β-D-xylopyranoside (for β-xylosidase), p-nitrophenyl-β-D-cellobioside (for β-cellobiosidase), p-nitrophenyl-β-*N*-acetylglucosamide (for NAGase), and p-nitrophenyl *N*,*N′*-diacetyl-β-D-chitobioside (for β-chitobiosidase). The activity of enzymes secreted by bacterial cells in the fecal environment was measured with the use of a reaction mixture containing 0.3 mL of the substrate solution (5 mM) and 0.2 mL of 1:10 (*v*/*v*) dilution of a fecal sample in 100 mM phosphate buffer (pH 7.0), which was centrifuged at 7211× *g* for 15 min. Incubation was carried out at 39 °C, and PNP was quantified at 400 nm (ONP was quantified at 420 nm) after the addition of 2.5 mL of 0.25 M-cold sodium carbonate. Enzyme activity was expressed in μmol of the product formed per hour per gram of fresh feces. To determine the total activity of selected fecal bacterial enzymes, including extracellular activity (see the procedure above) and intracellular activity, a sample of fecal matter diluted in phosphate buffer was mechanically disrupted by vortexing with glass beads (212–300 μm in diameter; four 1-min bursts, with 1-min cooling intervals on ice) in the FastPrep^®^-24 homogenizer (MP Biomedicals, Santa Ana, CA, USA). The resulting mixture was centrifuged at 7211× *g* for 15 min at 4 °C. The supernatant was used for the enzyme assay described above. Intracellular enzyme activity was calculated by comparing total enzyme activity with the activity of bacterial enzymes released into the fecal environment, and it was expressed in μmol of the product (PNP or ONP, respectively) formed per hour per gram of feces. The respective calculation formulas were derived based on the model curves for PNP and ONP (PNP or ONP standard solution in 100 mM phosphate buffer, pH 7.0, 40 mg/L). Extracellular enzyme activity was also expressed as a percentage of total enzyme activity (the release rate of enzymes).

### 2.5. Statistical Analysis

Data were checked for normality using the Shapiro–Wilk test and for homogeneity of variance using Levene’s test. When the assumptions of normality and homogeneity of variance were met, data were analyzed by one-way ANOVA followed by Tukey’s HSD post-hoc test. When variance homogeneity was violated, Welch’s ANOVA followed by the Games–Howell post-hoc test was applied. For variables that did not meet the assumptions of parametric analysis, the Kruskal–Wallis test followed by Dunn’s post-hoc test with Holm correction was used. The Benjamini–Hochberg adjustment was applied across the 44 overall tests of group differences for the enzyme-related outcomes. Differences between means were considered significant at *p* ≤ 0.05. All analyses were performed using R software (v.4.5.1) [21]. In the tables, data are presented as mean values ± standard deviation (SD).

## 3. Results

At the end of the experiment, the mean body weights were 4.25 ± 0.25, 4.24 ± 0.23, 4.28 ± 0.24, and 4.25 ± 0.23 kg in the control, CCM2wk, CCM4wk, and CCM6wk groups, respectively.

A significant decrease in the total, extracellular, and intracellular activities of fecal bacterial α-glucosidase was observed in all CCM groups. For β-galactosidase, extracellular and total activities were lower in all CCM groups, whereas intracellular activity was reduced only in the CCM4wk and CCM6wk groups (Table 3). The release rate of α-glucosidase was higher in the CCM4wk and CCM6wk groups than in the control group. The highest and lowest release rates of β-galactosidase were noted in CCM4wk and the control group, respectively. The extracellular bacterial activity of fecal β-glucosidase was higher in the CCM6wk, compared with the control group. The intracellular activity of β-glucosidase was highest in the control group and differed significantly from the CCM4wk and CCM6wk groups. Total β-glucosidase activity was significantly higher in the control group compared with the CCM4wk group. The release rate of β-glucosidase was higher in the CCM4wk and CCM6wk groups than in the control group. For α-galactosidase, extracellular activity was lower in all CCM groups than in the control group while intracellular and total activities were lowest in the CCM6wk group. The release rate of α-galactosidase was higher in all CCM groups than in the control group and was highest in the CCM6wk group. For β-glucuronidase, extracellular activity was significantly increased only in the CCM6wk group, while the intracellular activity was lower in all CCM groups than in the control group, where the release rate was lowest (*p* < 0.05 vs. all CCM groups). The extracellular and total activities of β-xylosidase remained unaffected by the applied dietary treatments. The lowest intracellular activity of bacterial β-xylosidase was observed in the CCM6wk group and was significantly lower than in the control and CCM2wk groups. The release rate of β-xylosidase increased in rabbits fed the CCM6wk diet (*p* < 0.05 vs. all other groups).

The total activity of bacterial α-arabinopyranosidase in the feces of rabbits was highest in the CCM6wk group and differed significantly from all remaining groups, whereas extracellular activity was higher in the CCM6wk group than in the control and CCM2wk groups, with the CCM4wk group showing an intermediate value (Table 4). The intracellular activity of α-arabinopyranosidase was lowest in the CCM2wk group and was significantly lower than in the control and CCM6wk groups. The release rate of α-arabinopyranosidase was highest in the CCM2wk group, which differed significantly from the control and CCM6wk groups. For α-arabinofuranosidase, extracellular activity was higher in the CCM6wk group than in the control and CCM2wk groups, with the CCM4wk group showing an intermediate value. Intracellular activity was higher in the CCM4wk and CCM6wk groups than in the CCM2wk group. Total activity was highest in the CCM6wk group and was higher than in the control and CCM2wk groups. The CCM2wk group showed the numerically highest release rate of α-arabinofuranosidase, but the difference did not reach statistical significance. The extracellular activity of β-cellobiosidase did not differ among groups. The intracellular activity of β-cellobiosidase was lowest in the CCM2wk group and was significantly lower than in the control and CCM6wk groups, whereas the CCM4wk group showed an intermediate value and differed significantly from the control group. Total β-cellobiosidase activity was higher in the CCM6wk group than in the CCM2wk group. The release rate of β-cellobiosidase was higher in the CCM2wk group than in the control group. Extracellular and total NAGase activities were higher in all CCM groups than in the control group. The intracellular activity of this enzyme increased in the feces of rabbits fed the CCM2wk diet, relative to the other groups. The feces of rabbits that received the CCM6wk diet exhibited a significantly elevated release rate of NAGase, compared with the control and CCM2wk groups (*p* < 0.05). Extracellular, intracellular, and total β-chitobiosidase activities were higher in all CCM groups than in the control group, with no significant differences among the CCM treatments. The release rate of β-chitobiosidase tended to be affected by dietary treatment, with the highest numerical value observed in the CCM6wk group.

As shown in Table 5, the digestibility coefficients of dry matter, organic matter, crude protein, ether extract, and gross energy did not differ among the groups (*p* > 0.05). In contrast, the digestibility of fiber fractions was markedly influenced by the duration of CCM feeding. Rabbits in the CCM4wk and CCM6wk groups were characterized by higher digestibility of NDF, ADF, and ADL, compared with both the control group and the CCM2wk treatment (*p* < 0.05). Notably, no differences in fiber digestibility were observed between the control group and the CCM2wk group, or between the CCM4wk and CCM6wk treatments.

## 4. Discussion

Crickets are a rich source of carbohydrates, proteins, lipids, minerals, and vitamins, which makes them a dietary ingredient that can exert a considerable influence on digestive processes through the action of endogenous and microbial enzymes [22]. Chitin is one of the most important components of the bodies of crickets and other insects. Chitin is not digested in the small intestine; therefore, its large quantities reach the large intestine of rabbits fed diets containing insect meal [23]. However, the amount of chitin reaching the hindgut and its disappearance were not directly quantified in the present study. Several studies have demonstrated that the cecal/colonic microbiota of humans and various animal species can degrade chitin and chitosan into smaller molecules that can be utilized by the host body [24]. Chitin degradation involves the release of chitinolytic enzymes from bacterial cells into the gut environment [25]. According to research, acidic chitinase is highly expressed in the stomach tissues of mice, chickens, and pigs, in addition to its microbial chitinolytic activity [6]. The present study showed that the duration of CCM feeding affected fecal bacterial enzyme activity and the apparent digestibility of detergent fiber fractions in adult rabbits. These effects were more evident after four and six weeks of dietary exposure, while two weeks of CCM feeding resulted in only minor changes in fiber digestibility. However, because gut microbial composition, microbial metabolites, and cecal fermentation parameters were not analyzed, these findings should not be interpreted as direct evidence of gut microbiota adaptation. Rather, they indicate changes in fecal bacterial enzymatic activity occurring in response to CCM feeding. Both exochitinases analyzed in this study catalyze the progressive release of acetylchitobiose or N-acetylglucosamine from the non-reducing end of chitin and are referred to as β-chitobiosidase and NAGase, respectively [26]. In the current experiment, the activity of both NAGase and chitobiosidase was markedly increased by the addition of CCM to rabbit diets. Furthermore, varying the time of CCM administration was found to significantly affect the release rates of the studied enzymes, as well as their total and extracellular activities. The release rates of both enzymes increased gradually with prolonged CCM administration. In the case of NAGase, this was due to its diminished total activity in groups fed CCM for four or six weeks vs. those fed CCM for two weeks, along with similar levels of its extracellular activity. As for chitobiosidase, an increase in its extracellular activity was noted with prolonged CCM administration. In turn, the total activity of this enzyme was comparable in all three CCM treatments. This is an example of a different mechanism leading to enhanced extracellular chitinolytic activity through an increase in the release rate of the enzyme. Microorganisms play a key role in fermentation processes in the rabbits’ ceca [27]. The chemically complex macromolecules that constitute a sizable portion of nutrients bypassing the upper gastrointestinal tract are too large to be transferred across the bacterial cytoplasmic membrane [28]. Before being absorbed by microbial cells, they must be hydrolyzed into small particles by extracellular enzymes. Therefore, extracellular enzymes are important variables influencing the utilization of organic matter in the large gut ecosystem [29]. There is no doubt that the release rates of enzymes from bacterial cells into the cecal environment are influenced by various factors, including the counts of microbes, their physiology and activity, as well as the presence of potential substrates and bioactive compounds in the cecal/colonic milieu [30].

The extracellular activity of major glycolytic enzymes, i.e., α-glucosidase, α- and β-galactosidase, decreased in response to the dietary inclusion of CCM, with a concomitant increase in their release rates. These changes became evident after only two weeks of feeding the CCM diet, and persisted as the feeding period was extended to four and six weeks. Interpretation of these results requires caution because CCM inclusion was accompanied by changes in the proportions of alfalfa meal, corn, barley, and other dietary ingredients. Therefore, the observed changes in fecal glycosidase activity may reflect not only the presence of insect-derived chitin, but also differences in starch supply and fiber origin. Consequently, these effects should be interpreted as a response to the complete CCM-containing diet rather than as a chitin-specific effect. β-glucosidase is widespread among gut microbiota, and it may play an important role in the release of active (e.g., antioxidant) molecules from plant metabolites (e.g., polyphenols), thus enhancing the bioavailability of health-promoting dietary ingredients [31]. On the other hand, under specific conditions, microbial β-glucosidase may be responsible for an undesirable increase in toxin and xenobiotic uptake from the intestines into the bloodstream [32]. α-galactosidase is an exoglycosidase that targets branched polysaccharides, including galactomannans and galacto-glucomannans, as well as galactooligosaccharides such as raffinose, melibiose, and stachyose [33]. Microbial α-arabinopyranosidase, α-arabinofuranosidase, and β-cellobiosidase play a key role in the hydrolysis of arabinoxylans and lignocellulosic plant cell wall components in the large intestine [34].

It has been reported that dietary insect products may provide natural antibiotic and anti-pathogenic substances, thus modulating the microbial community in the intestines towards a healthier state by promoting the growth of beneficial bacteria [35]. In this context, chitosan derived from crickets was found to serve as a prebiotic promoting probiotic bacteria and restricting pathogens [22]. β-glucuronidase is an enzyme produced mostly by unbeneficial gut bacteria, and a high level of β-glucuronidase activity may disrupt the body’s ability to detoxify natural hormones, potentially harmful metabolites, and environmental chemicals [36]. In the present study, the dietary inclusion of CCM in rabbit diets did not enhance the total activity of fecal β-glucuronidase; however, it increased the extracellular activity of this enzyme irrespective of feeding duration. This may indicate that the addition of CCM did not promote the growth of the undesirable bacterial population and that the increase in the extracellular activity of β-glucuronidase could be attributed to differences in fat source and fatty acid composition and thus to differences in the amount of glucuronidated bile acid derivatives [37,38]. It should be noted, however, that in the present experiment, almost no differences in ether extract digestibility were found between the groups. In contrast, the digestibility coefficients of NDF, ADF, and ADL were significantly higher in the groups fed CCM diets for four and six weeks. The increase in the values of these coefficients was accompanied by changes in the activity and release rate of selected bacterial enzymes, particularly β-cellobiosidase, NAGase, and β-chitobiosidase. This association indirectly suggests a functional link between the fecal enzymatic profile and the utilization of detergent fiber fractions that may include insect-derived structural components, including chitin. The increased activities of NAGase and β-chitobiosidase are consistent with enhanced chitinolytic potential; however, chitin degradation was not measured directly. Moreover, the higher digestibility of NDF, ADF, and ADL may also have been influenced by differences in starch supply and fiber origin between the diets. According to the European Food Safety Authority (EFSA) Panel on Nutrition, Novel Foods and Food Allergens (NDA) [39], the chitin content of insect-based products is often measured as ADF. However, this method leads to an overestimation of chitin content since the ADF fraction contains significant amounts of amino acids. Therefore, chitin content is probably lower than reported. According to Hahn et al. [40], ADF-ADL represents a compromise between accuracy and equipment demand and is a suitable method for determining the chitin content of both insects and their residues.

The digestibility of nutrients and energy in rabbits fed a diet with the addition of crickets has been previously evaluated by Volek et al. [16]. They found no significant differences in ADF digestibility between the control group and the experimental group fed a diet containing *Acheta domesticus*. However, energy digestibility decreased in the latter group. It should be stressed that in the cited study, the inclusion level of crickets was lower (3%), the experiment involved young growing rabbits (after weaning), digestibility was determined between experimental days 18 and 22 (i.e., between 50 and 54 days of age), and the digestibility of NDF and ADL was not measured. In turn, in the present study, rabbits fed CCM diets for four and six weeks exhibited higher digestibility of NDF, ADF, and ADL, compared with both the control group and the CCM2wk group, whereas no differences were observed between the control group and the CCM2wk group or between the CCM4wk and CCM6wk treatments (Table 5). This indicates that fiber utilization improved only after a longer feeding period, with measurable changes becoming evident after four weeks of CCM exposure, although this should be interpreted as a duration-dependent enzymatic response rather than direct evidence of a defined microbiota adaptation timeline. The increased fiber digestibility observed after longer CCM feeding periods was accompanied by a marked increase in the activity of fecal NAGase and β-chitobiosidase, as well as by enhanced extracellular activity of several cellulolytic and hemicellulolytic enzymes (Table 3 and Table 4). The improvement in ADF and ADL digestibility is particularly noteworthy, as these fractions include chitin measured analytically within detergent fiber residues. It should be noted that the apparent digestibility of ADL does not reflect the degradation of true plant lignin, but may partly result from the disappearance of resistant nitrogen-containing fractions of the insect cuticle recovered in the ADL residue. This supports previous observations that insect-derived chitin contributes substantially to the ADF fraction and is progressively degraded by adapted microbial communities [41]. Notably, the absence of differences in gross energy digestibility among treatments suggests that the primary nutritional impact of prolonged CCM feeding was directed towards fiber utilization rather than macronutrient digestion. This may indicate a targeted microbial response towards complex polysaccharides, without influencing overall nutrient digestibility.

The present study has several limitations that should be acknowledged. First, as noted above, fecal enzyme activity was used as an indirect indicator of hindgut microbial function. Hard feces are formed during colonic passage, which involves fluid and particle separation [42,43], and their enzymatic profile may also be affected by oxygen exposure during defecation and the reduced viability of obligate anaerobes. Therefore, enzyme activities measured in hard feces may not quantitatively reflect microbial activity or fermentation processes in the cecum. Second, chitin degradation was not measured directly, and detergent fiber fractions provide only indirect information in this respect. Third, CCM inclusion was accompanied by changes in cereal inclusion and fiber source, preventing the independent effects of chitin, starch, and plant-derived fiber from being distinguished. Finally, adult rabbits were used to reduce variability associated with growth, weaning, and post-weaning maturation of the digestive tract. However, this limits the extrapolation of the results to weaned or growing rabbits, whose cecal microbiota is still developing and which are more directly relevant to commercial meat production. At the same time, the use of adult rabbits is also relevant from a practical perspective, as many rabbits are kept as companion animals and dietary strategies supporting gastrointestinal function may be important in this population.

## 5. Conclusions

In conclusion, feeding adult rabbits a diet containing Cuban cricket meal was associated with changes in fecal bacterial enzyme activity and increased the apparent digestibility of NDF, ADF, and ADL after four and six weeks of exposure. The response involved changes in the activity and release rate of selected enzymes related to the hydrolysis of complex carbohydrates and chitin-derived substrates. These findings provide indirect evidence of a duration-dependent functional enzymatic response to CCM feeding; however, because the diets also differed in cereal inclusion and fiber source, the specific contribution of CCM-derived chitin cannot be determined. Because the study involved adult rabbits with an established cecal microbiota, neither the apparent four-week response period nor any inference regarding dietary safety can be directly extrapolated to weaned or growing rabbits.

## Figures and Tables

**Table 1:** Ingredient composition of rabbit diets (%).

Ingredient	Diet	
	Control Diet	Cuban Cricket Diet
Soybean meal (SBM)	14	-
Cuban cricket meal (CCM)	-	10
Alfalfa meal	22	17
Corn	26.5	30
Barley	14	19
Wheat bran	10	11
Rapeseed oil	1.5	-
Arbocel	8	9
Salt	1	1
Ground limestone	2	2
Vitamin-mineral premix	1	1

ARBOCEL^®^ A300 powdered cellulose (JRS PHARMA GmbH & Co. KG, Rosenberg, Germany; average particle size 320 µm).

**Table 2:** Chemical composition (% of fresh matter) and measured energy content (MJ/kg) of soybean meal, Cuban cricket meal, and rabbit diets.

	Soybean Meal (SBM)	Cuban Cricket Meal (CCM)	Diets	
			Control Diet	Cuban Cricket Diet
Dry matter	89.0	92.5	88.8	88.8
Crude ash	6.2	5.4	5.8	5.7
Crude protein	46.3	63.5	16.13	16.06
Ether extract	2.0	16.5	3.83	3.89
Calculated starch	1.60	0.00	24.69	29.23
Neutral detergent fiber	14.5	17.28	24.53	25.16
Acid detergent fiber	7.35	10.9	13.69	13.72
Acid detergent lignin	1.42	4.94	1.31	1.74
Gross energy	18.64	22.37	16.78	16.89

Starch contents were calculated based on ingredient composition and average values reported in the INRAE-CIRAD-AFZ Feed Tables [18]. The starch contribution of CCM was assumed to be negligible.

**Table 3:** Activity of bacterial α-glucosidase, β-glucosidase, α-galactosidase, β-galactosidase, β-glucuronidase, and β-xylosidase in the feces of rabbits fed a control diet and a diet containing Cuban cricket meal (CCM) for six weeks; the latter diet was provided during the final two or four weeks or during the entire six-week feeding period.

	Control Diet	CCM2wk	CCM4wk	CCM6wk	*p*-Value
α-glucosidase *					
Extracellular	7.64 ± 1.17 ^a^	5.81 ± 0.96 ^b^	5.76 ± 0.96 ^b^	5.57 ± 1.33 ^b^	0.004
Intracellular	4.02 ± 1.29 ^a^	1.96 ± 0.65 ^b^	1.65 ± 0.55 ^b^	1.25 ± 0.52 ^b^	0.001
Total	11.66 ± 1.69 ^a^	7.77 ± 1.38 ^b^	7.41 ± 1.22 ^b^	6.82 ± 1.31 ^b^	<0.001
Release rate ^#^	65.89 ± 8.18 ^b^	75.03 ± 5.90 ^ab^	77.80 ± 5.28 ^a^	81.21 ± 8.89 ^a^	0.002
β-glucosidase *					
Extracellular	2.19 ± 0.70 ^b^	2.49 ± 0.55 ^ab^	2.78 ± 0.69 ^ab^	3.22 ± 0.75 ^a^	0.038
Intracellular	3.16 ± 1.46 ^a^	1.46 ± 0.58 ^ab^	0.95 ± 0.36 ^b^	0.83 ± 0.37 ^b^	0.001
Total	5.35 ± 1.52 ^a^	3.95 ± 0.91 ^ab^	3.74 ± 0.95 ^b^	4.05 ± 0.77 ^ab^	0.027
Release rate ^#^	41.93 ± 12.44 ^b^	63.81 ± 9.31 ^ab^	74.96 ± 5.87 ^a^	79.26 ± 9.65 ^a^	<0.001
α-galactosidase *					
Extracellular	23.66 ± 4.46 ^a^	17.43 ± 2.67 ^b^	15.39 ± 1.95 ^b^	13.99 ± 1.90 ^b^	<0.001
Intracellular	22.24 ± 6.00 ^a^	8.09 ± 2.23 ^b^	5.31 ± 2.57 ^bc^	3.02 ± 1.27 ^c^	<0.001
Total	45.90 ± 8.54 ^a^	25.52 ± 4.30 ^b^	20.70 ± 3.44 ^bc^	17.00 ± 1.47 ^c^	<0.001
Release rate ^#^	51.91 ± 6.74 ^c^	68.59 ± 4.72 ^b^	75.16 ± 9.22 ^ab^	82.16 ± 7.57 ^a^	<0.001
β-galactosidase *					
Extracellular	39.28 ± 6.46 ^a^	22.88 ± 4.72 ^b^	22.32 ± 3.79 ^b^	21.21 ± 3.95 ^b^	<0.001
Intracellular	29.75 ± 8.16 ^a^	13.57 ± 4.79 ^ab^	9.17 ± 4.76 ^b^	9.03 ± 2.68 ^b^	0.001
Total	69.03 ± 9.44 ^a^	36.45 ± 7.19 ^b^	31.49 ± 7.30 ^b^	30.24 ± 5.92 ^b^	<0.001
Release rate ^#^	57.35 ± 9.05 ^b^	63.16 ± 8.69 ^ab^	72.24 ± 8.87 ^a^	70.42 ± 5.07 ^ab^	0.016
β-glucuronidase *					
Extracellular	20.48 ± 3.96 ^b^	25.89 ± 4.07 ^ab^	25.00 ± 4.83 ^ab^	27.93 ± 4.48 ^a^	0.018
Intracellular	21.50 ± 3.86 ^a^	12.33 ± 1.45 ^b^	12.37 ± 2.66 ^b^	10.75 ± 4.80 ^b^	<0.001
Total	41.99 ± 6.78	38.22 ± 4.42	37.36 ± 5.85	38.68 ± 3.02	0.295
Release rate ^#^	48.76 ± 4.48 ^b^	67.52 ± 3.92 ^a^	66.80 ± 5.67 ^a^	72.41 ± 11.43 ^a^	0.001
β-xylosidase *					
Extracellular	1.73 ± 0.53	2.23 ± 0.72	1.72 ± 0.42	1.86 ± 0.72	0.332
Intracellular	1.85 ± 0.76 ^a^	2.03 ± 1.00 ^a^	1.50 ± 0.65 ^ab^	0.86 ± 0.52 ^b^	0.006
Total	3.59 ± 1.17	4.25 ± 1.54	3.22 ± 0.75	2.72 ± 1.06	0.114
Release rate ^#^	49.11 ± 9.36 ^b^	53.77 ± 8.43 ^b^	54.18 ± 12.08 ^b^	68.34 ± 10.88 ^a^	0.007

* Extracellular, intracellular, and total enzyme activities are expressed as µmol of product formed per hour per gram of fresh feces (µmol/h/g fresh feces); ^#^ extracellular activity expressed as a percentage of total (extra- + intracellular) enzyme activity (%); ^a–c^ Mean values within rows with no common superscripts are significantly different at *p* ≤ 0.05.

**Table 4:** Activity of bacterial α-arabinopyranosidase, α-arabinofuranosidase, β-cellobiosidase, NAGase, and β-chitobiosidase in the feces of rabbits fed a control diet and a diet containing Cuban cricket meal (CCM) for six weeks; the latter diet was provided during the final two or four weeks or during the entire six-week feeding period.

	Control Diet	CCM2wk	CCM4wk	CCM6wk	*p*-Value
α-arabinopyranosidase *					
Extracellular	1.05 ± 0.41 ^b^	1.00 ± 0.15 ^b^	1.27 ± 0.30 ^ab^	1.73 ± 0.52 ^a^	0.002
Intracellular	0.88 ± 0.29 ^ab^	0.27 ± 0.15 ^c^	0.52 ± 0.22 ^bc^	1.09 ± 0.46 ^a^	<0.001
Total	1.92 ± 0.62 ^b^	1.27 ± 0.18 ^b^	1.79 ± 0.48 ^b^	2.82 ± 0.95 ^a^	0.001
Release rate	52.98 ± 13.12 ^c^	79.43 ± 10.15 ^a^	71.76 ± 6.68 ^ab^	62.20 ± 4.83 ^bc^	<0.001
α-arabinofuranosidase *					
Extracellular	0.95 ± 0.10 ^b^	1.18 ± 0.36 ^b^	1.77 ± 0.70 ^ab^	2.02 ± 0.42 ^a^	<0.001
Intracellular	0.89 ± 0.50 ^ab^	0.51 ± 0.23 ^b^	1.48 ± 0.75 ^a^	1.84 ± 0.46 ^a^	0.001
Total	1.84 ± 0.55 ^bc^	1.69 ± 0.42 ^c^	3.25 ± 1.39 ^ab^	3.87 ± 0.76 ^a^	0.001
Release rate ^#^	56.11 ± 17.32	69.41 ± 11.40	55.73 ± 7.50	52.52 ± 6.28	0.060
β-cellobiosidase *					
Extracellular	1.07 ± 0.31	1.11 ± 0.24	1.12 ± 0.38	1.44 ± 0.46	0.232
Intracellular	0.86 ± 0.24 ^a^	0.23 ± 0.12 ^c^	0.42 ± 0.16 ^bc^	0.64 ± 0.42 ^ab^	0.001
Total	1.93 ± 0.42 ^ab^	1.33 ± 0.25 ^b^	1.54 ± 0.44 ^ab^	2.09 ± 0.60 ^a^	0.010
Release rate ^#^	55.01 ± 10.29 ^b^	83.20 ± 8.54 ^a^	72.54 ± 8.29 ^ab^	69.69 ± 13.31 ^ab^	0.002
NAGase ^&^					
Extracellular	1.91 ± 0.69 ^b^	6.29 ± 0.89 ^a^	6.55 ± 1.17 ^a^	6.83 ± 1.06 ^a^	<0.001
Intracellular	1.02 ± 0.35 ^b^	3.90 ± 1.31 ^a^	1.79 ± 0.65 ^b^	1.20 ± 0.55 ^b^	0.001
Total	2.93 ± 0.77 ^b^	10.18 ± 2.10 ^a^	8.34 ± 1.17 ^a^	8.03 ± 0.81 ^a^	<0.001
Release rate ^#^	64.41 ± 12.76 ^bc^	62.56 ± 5.15 ^c^	78.37 ± 7.40 ^ab^	84.79 ± 7.65 ^a^	0.001
β-chitobiosidase *					
Extracellular	0.93 ± 0.20 ^b^	4.24 ± 1.49 ^a^	5.18 ± 1.11 ^a^	5.51 ± 1.17 ^a^	<0.001
Intracellular	0.49 ± 0.11 ^b^	3.13 ± 1.36 ^a^	2.51 ± 1.50 ^a^	2.06 ± 0.68 ^a^	<0.001
Total	1.42 ± 0.23 ^b^	7.37 ± 2.14 ^a^	7.69 ± 1.94 ^a^	7.57 ± 1.19 ^a^	<0.001
Release rate ^#^	65.46 ± 7.43	57.60 ± 12.64	68.99 ± 12.80	72.82 ± 8.86	0.056

^&^ NAGase, N-acetyl-β-D-glucosaminidase; * Extracellular, intracellular, and total enzyme activities are expressed as µmol of product formed per hour per gram of fresh feces (µmol/h/g fresh feces); ^#^ extracellular activity expressed as a percentage of total (extra- + intracellular) enzyme activity (%); ^a–c^ Mean values within rows with no common superscripts are significantly different at *p* ≤ 0.05.

**Table 5:** Nutrient digestibility in rabbits fed a control diet and a diet containing Cuban cricket meal (CCM) for six weeks; the latter diet was provided during the final two or four weeks or during the entire six-week feeding period.

	Control Diet	CCM2wk	CCM4wk	CCM6wk	*p*-Value
Digestibility (%)					
Dry matter	64.50 ± 3.98	65.22 ± 2.28	67.61 ± 3.08	66.07 ± 1.80	0.191
Organic matter	67.58 ± 2.98	68.24 ± 2.98	69.35 ± 3.91	69.36 ± 2.25	0.590
Crude protein	72.29 ± 1.69	72.12 ± 1.82	71.95 ± 1.64	71.63 ± 1.29	0.869
Ether extract	90.07 ± 0.31	90.40 ± 0.42	90.14 ± 0.24	90.08 ± 0.29	0.278
Neutral detergent fiber	33.93 ± 3.47 ^b^	35.46 ± 2.59 ^b^	41.07 ± 1.77 ^a^	41.67 ± 2.94 ^a^	<0.001
Acid detergent fiber	24.14 ± 2.21 ^b^	24.27 ± 2.69 ^b^	27.91 ± 1.98 ^a^	28.09 ± 2.95 ^a^	0.002
Acid detergent lignin	24.29 ± 3.96 ^b^	24.12 ± 2.41 ^b^	31.32 ± 2.42 ^a^	31.24 ± 3.21 ^a^	<0.001
Gross energy	65.24 ± 3.34	66.89 ± 1.85	67.39 ± 2.81	67.02 ± 1.31	0.327

^a,b^ Mean values within rows with no common superscripts are significantly different at *p* ≤ 0.05.

## Data Availability

The data presented in this study are available upon request from the corresponding author.
